# Transvaginal two-dimensional ultrasound measurement of cervical volume to predict the outcome of the induction of labour: a prospective observational study

**DOI:** 10.1186/s12884-021-03929-9

**Published:** 2021-06-22

**Authors:** S. R. Athulathmudali, M. Patabendige, S. K. Chandrasinghe, P. H. P. De Silva

**Affiliations:** 1 North Colombo Teaching Hospital, Ragama, Sri Lanka; 2 Castle Street Hospital for Women, Colombo, Sri Lanka

**Keywords:** Induction of labour, Bishop Score, Cervical volume, 2D Ultrasound, Successful induction

## Abstract

**Background:**

Assessing the likelihood of success of induction of labour using ultrasonically measured cervical volume is an important research question.

**Method:**

A prospective observational study was carried out at North Colombo Teaching Hospital, Ragama, Sri Lanka. Pre-induction digital cervical assessment, transvaginal cervical length, and cervical volume measurements were performed. Inductions with singleton pregnancies at term were included. Basic demographic and clinical details, independent variables (Bishop score, cervical length and cervical volume), and dependent variables (frequency of delivery within 24 h and induction to delivery interval) were recorded. Vaginal delivery within 24 h was the primary outcome.

**Results:**

We studied 100 pregnant women who had induction of labour. Median (IQR) Bishop score was 5 (3–6), mean (SD) cervical length was 3.6 (0.7) cm, and mean (SD) cervical volume was 27.5 (10.4) cm^3^. Cervical length was the best predictor for predicting the likelihood of vaginal delivery within 24 h [aOR – 12.12 (3.44, 42.71); < 0.001], and cervical volume also appeared to be a significant potential predictor [aOR-1.10 (1.01, 1.17); 0.01]. Cervical length was found to have the highest AUC (0.83) followed by the cervical volume (0.74). The best cut-off value for cervical volume in predicting the likelihood of vaginal delivery within 24 h was less than 28.5 cm^3^ with a sensitivity of 72% and specificity of 74%.

**Conclusions:**

Transvaginal sonographic measurement of cervical volume appears to be a potential novel predictor for the likelihood of vaginal delivery within 24 h of induction of labour. Cervical length is still more superior to cervical volume in predicting the likelihood of vaginal delivery. Bishop score was not a significant predictor in this context.

## Introduction

Induction of labour is one of the most frequently performed obstetric interventions in current obstetric practice [1]. The digital cervical assessment with Bishop score is subjective and several studies have shown a poor predictive value for the outcome of induction [2, 3]. Several recent studies have demonstrated that transvaginal sonographic assessment of the cervical length can yield a more sensitive prediction of successful induction, compared to Bishop scoring [4–10]. Cochrane review on ‘methods for assessing pre‐induction cervical ripening’ has concluded that there is insufficient evidence to support the use of transvaginal sonography over the standard digital vaginal assessment in pre‐induction cervical ripening [11]. Therefore, the likelihood of impact from measuring the cervical volume to assess cervical favourability is an important area to be studied.

Cervical volume calculation includes both the length and the diameter of the cervix which might have the potential to cover two aspects of cervical scoring systems [cervical length and Bishop score]. There is a dearth of literature with regards to the cervical volume to assess pre-induction cervical ripening. The primary aim was to evaluate the association between ultrasonically measured cervical volume and the frequency of delivery within 24 h. The secondary aim was to compare the frequency of delivery within 24 h with ultrasonically measured cervical length and Bishop score.

## Materials and method

### Study design and setting

A prospective observational study was carried out at an obstetric unit, North Colombo Teaching Hospital (NCTH), Ragama, Sri Lanka. Eligible pregnant women were selected after hospital admission for the elective induction of labour. All the consecutive inductions performed over an 8 weeks period were assessed for eligibility.

### Inclusion and exclusion criteria

They were singleton pregnancies with vertex presentation at term. Women with normal booking body mass index (BMI), normal weight gain during pregnancy, and those with an unfavourable cervix (Bishop score less than 6) at the time of hospital admission were included. These women were induced electively after 37 weeks of gestation. Indications such as past delivery date, pregnancy-induced hypertension without evidence of fetal compromise, and well-controlled gestational diabetes mellitus with adequate fetal growth were included in the study.

Women with previous uterine scar including caesarean delivery or myomectomy, multiple pregnancies, malpresentation, pre-labour rupture of membranes, evidence of fetal compromise, history of previous cervical surgeries, Bishop score more than 6 at the time of hospital admission and women with established labour were excluded from the study.

### Sonographic cervical volume assessment

The pre-induction cervical assessment was done using both the Bishop score and transvaginal ultrasonography. Sonographic assessment in all cases included was done by the first author (SRA). The procedure was standardized according to the recommendations for cervical length assessment published by the International Society of Ultrasound in Obstetrics and Gynecology (ISUOG) [12]. Transvaginal ultrasound (TVS) was performed with a sector phased array of 7 MHz probes (Samsung Medison Co.Ltd-Korea), according to the ISUOG practice advice [12]. The appropriate 1-day training was obtained prior to the study from an accredited sonographer in this field. Women were asked to empty the bladder before transvaginal ultrasound examination. Digital cervical assessment using Bishop score was performed by the treating physicians in the unit. The sonographer was blinded to the findings of the digital cervical assessment.

Three measurements were taken and the shortest measurement was taken as the final cervical length. Measurement of the anteroposterior diameter of the cervix was obtained at the midpoint of the cervix, right-angled to the endocervical canal. Cervical volume was calculated assuming the cervix as a cylinder in geometric view (V = πr^2^h).

### Protocol for induction of labour

Induction of labour was performed according to the local unit protocol which is based on the National Institute of Clinical Excellence (NICE) clinical guideline for induction of labour and the guideline prepared by the Sri Lanka College of Obstetricians Gynaecologists [13, 14]. After a morning dose of Dinoprostone 3 mg (prostaglandin E_2_ vaginal tablet), women were assessed after 6 h. Depending on the findings, insertion of another Dinoprostone 3 mg tablet (maximum of two tablets giving rise to one cycle) was placed if the cervix was unfavourable (Bishop score less than 6). If the cervix was favourable (Bishop score more than 6), women were scheduled for amniotomy on the next day morning. Oxytocin augmentation was started if uterine contractions were inadequate after 2 h of observation after amniotomy in the labour ward.

Induced women were allowed to progress if labour started before the scheduled amniotomy. Women who proceeded into labour before the scheduled amniotomy after prostaglandins were augmented with oxytocin, only if their progress was poor in due course of labour. All women were offered intrapartum continuous CTG monitoring. If induction has not been successful with one cycle of Dinoprostone, women were managed according to the local unit protocol which consisted of three options: another cycle of induction with prostaglandins, insertion of transcervical Foley catheter for a maximum of 48 h and caesarean delivery.

### Outcome measures

Independent variables (test variables) were cervical volume, cervical length, and Bishop score. Dependent variables (outcomes) were frequency of delivery within 24 h and induction to delivery interval. Baseline variables (possible confounders) were parity and maternal age. The primary outcome measure was the frequency of delivery within 24 h.

### Statistical analysis

Means and standard deviations were used to summarise numerical data where relevant. Correlation between induction to delivery interval and Bishop score, cervical length, and cervical volume were calculated using Pearson correlation coefficient. Cut-off levels for the Bishop score, cervical length, and cervical volume to predict the likelihood of delivery within 24 h of induction were calculated using a Receiver Operating Characteristics (ROC) curve and Youden index that maximizes the vertical distance from the line of equality (reference line) to the left upper point of the respective curve. Logistic regression analysis was used to correlate those who delivered vaginally within 24 h with cervical volume, cervical length, and Bishop score while controlling for confounders such as BMI, parity, maternal age, oxytocin augmentation, repeat prostaglandin use, and gestational age at induction. *P*-value < 0.05 was taken as the significant level. Ethical approval was obtained from the Ethics Review Committee, Faculty of Medicine, University of Kelaniya, Sri Lanka.

## Results

### Demographic and clinical details

We studied 100 pregnant women who underwent induction of labour consecutively during the study period. The mean (range) body mass index at booking was 24.0 (12.1) m^2^/kg. 71 (71%) were nulliparous and 48 (48%) women had delivered within 24 h following induction. There were 20 (20%) caesarean deliveries and all of them had failed induction as the indication. Table 1 shows baseline characteristics of the study participants and Table 2 shows outcomes after induction of labour.

**Table 1:** Characteristics and indications of the study participants (*N* = 100)

**Characteristic**	**Nulliparous women** ***n* = 71 (71.0%)**	**Parous women** ***n* = 29 (29.0%)**
**Maternal age in years: mean (SD)**	27.2 (4.2)	31.3 (4.4)*
**Gestational age at induction of labour in days: mean (SD)**	278.7 (9.6)	282.9 (6.5)
**Indications for induction**		
**- Past delivery date, n (%)**	36 (50.7)	25 (86.2)
**- Pregnancy-induced hypertension, n (%)**	6 (8.5)	None
**- Gestational diabetes mellitus, n (%)**	29 (40.8)	4 (13.8)
**Bishop score: median (IQR)**	5 (3–6)	5 (4–7)
**Cervical length in cm: mean (SD)**	3.6 (0.7)	3.5 (0.9)
**Cervical volume in cm**^**3**^**: mean (SD)**	28.6 (11.3)	24.8 (7.6)

*SD* Standard deviation, *IQR* Interquartile range

^*^p-value < 0.05

**Table 2:** Characteristics and outcomes after induction of labour (*N* = 100)

**Characteristic**	**Nulliparous women** ***n* = 71 (71%)**	**Parous women** ***n* = 29 (29%)**
**Mode of delivery**		
**- Spontaneous vaginal**	47 (66.2)	26 (89.7)
**- Instrumental**	7 (9.9)	None
**- Caesarean**	17 (23.9)	3 (10.3)
**Oxytocin augmentation, n (%)**	53 (74.6)	8 (27.6)
**Uterine hyperstimulation**^**a**^	11 (15.5)	None
**Induction to delivery interval in hours: mean (SD)**	40.8 (27.8)	17.4 (21.2)*
**Birthweight: mean (SD)**	3.1(0.6)	3.0 (0.3)
**SCBU admission, n (%)**	3 (4.2)	2 (6.9)
**Need for a second tablet of Dinoprostone 3 mg, n (%)**	36 (50.7)	4 (13.8)

*SD* Standard deviation, *SCBU* Special care baby unit

^*^p-value < 0.05

^a^ more than five contractions in 10 min with changes in fetal heart rate (defined as a non-reassuring cardiotocograph by the treating physician)

### Correlations between test variables and induction to delivery interval

Median (IQR) Bishop score was 5 (3–6), mean (SD) cervical length was 3.6 (0.7) cm, and mean (SD) cervical volume was 27.5 (10.4) cm^3^. There was a significant negative correlation between induction to delivery interval and the Bishop score (Pearson correlation = 0.3, *p* < 0.01). There was a significant positive correlation between induction to delivery interval and the sonographically assessed cervical length (Pearson correlation = 0.445, *p* < 0.01) and cervical volume (Pearson correlation = 0.368, *p* < 0.01).

### Associations of dependent and independent variables in predicting the likelihood of vaginal delivery within 24 h

Table 3 summarises logistic regression analysis showing associations of dependent and independent variables in predicting the likelihood of vaginal delivery within 24 h. The cervical length has shown to be the best parameter for predicting the likelihood of vaginal delivery within 24 h [aOR – 12.12 (3.44, 42.71); < 0.001]. Association of the cervical volume was also significant for predicting the likelihood of vaginal delivery within 24 h [aOR-1.10 (1.01, 1.17); 0.01]. Bishop score was not shown to be significant [aOR-1.04 (0.79, 1.38); 0.76].

**Table 3:** Logistic regression analysis showing associations of outcome variables for predicting likelihood of vaginal delivery within 24 h

**Variable**	**Likelihood of vaginal delivery within 24 h**	
	**Crude OR (95% CI); *p* value**	**aOR (95% CI); *p* value**
**Bishop score**	0.78 (0.63, 0.97); 0.03	1.04 (0.79, 1.38); 0.76
**Cervical length (cm)**	11.19 (4.12, 30.44); < 0.001	12.12 (3.44, 42.71); < 0.001
**Cervical volume (cm**^**3**^**)**	1.10 (1.05, 1.16); < 0.001	1.10 (1.01, 1.17); 0.01

*OR* Odds Ratio, *aOR* Adjusted Odds Ratio, *CI* Confidence Interval

**Fig. 1:**
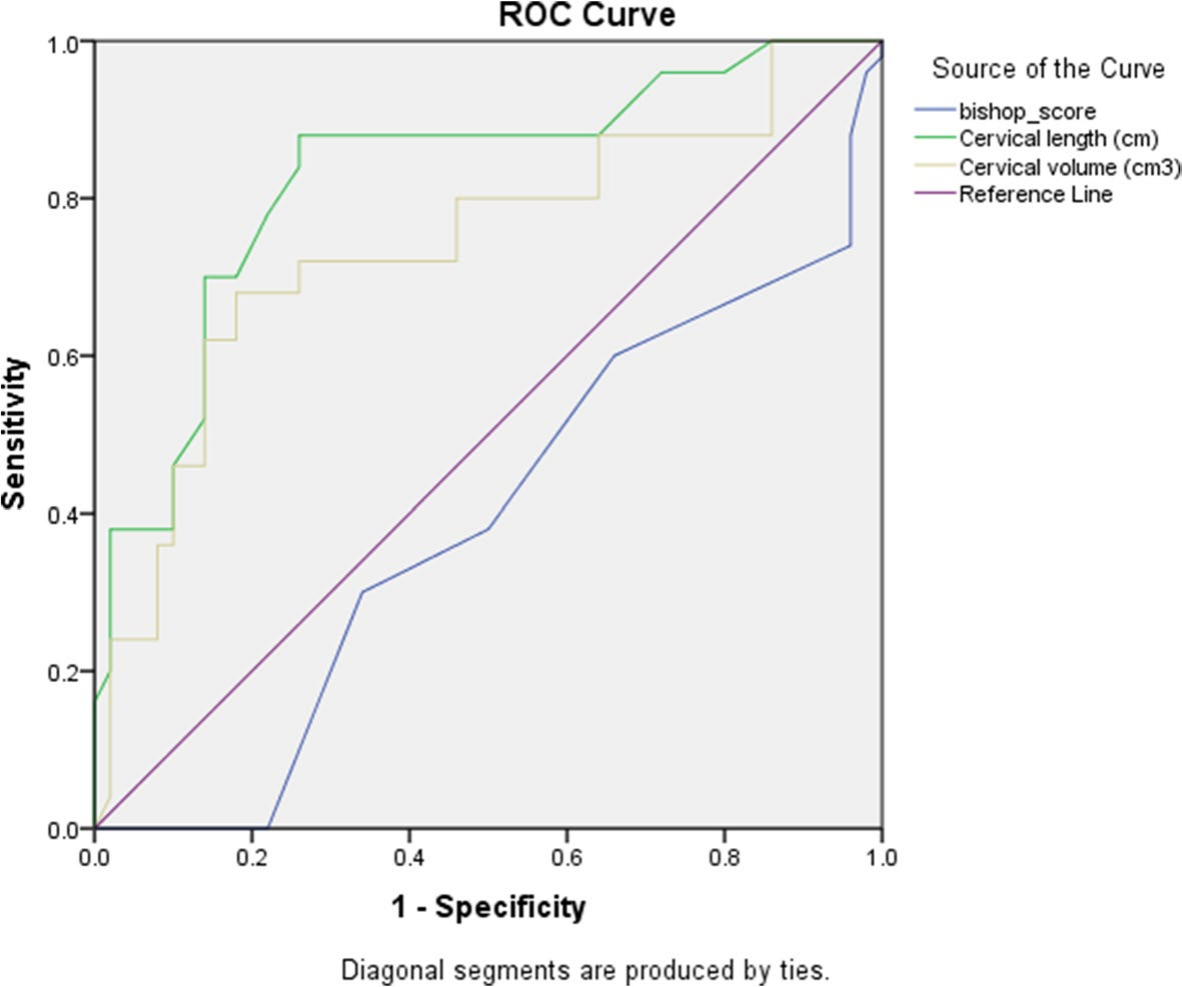
Receiver Operating Characteristic curves for sonographically measured cervical length (cm), cervical volume (cm^3^) and Bishop score in predicting the likelihood of delivery within 24 h

Figure 1 displays the ROC curves for sonographically measured the cervical length, cervical volume, and Bishop score for predicting the likelihood of delivery within 24 h. The curves constructed for sonographically measured cervical length and cervical volume was above the 45° line, indicating a significant relationship with the likelihood of delivery within 24 h. The area under the curve (AUC) of ROC curves for Bishop score in predicting the likelihood of vaginal delivery within 24 h was 0.39 (0.28—0.50); *p* = 0.06. AUC for cervical length was 0.83 (0.74—0.91); *p* < 0.001 and AUC for cervical volume was 0.74 (0.64—0.84); *p* < 0.001. The best cut-off values in predicting the likelihood of delivery within 24 h for cervical length was less than 3.71 cm (sensitivity of 88% and a specificity of 74%), for cervical volume was less than 28.5 cm^3^ (sensitivity of 72% and specificity of 74%) and for Bishop score was more than 4.5 (sensitivity of 62% and specificity of 50%).

## Discussion

This prospective observational study assessed the ability of ultrasonically measured cervical volume, cervical length and Bishop score to predict the outcome of induction of labour. Investigators tested the cervical volume as a proposed novel way of predicting the success of induction of labour. In the results, the cervical volume was found to be significant in predicting vaginal delivery within 24 h after induction of labour. But, cervical length is still far superior to the cervical volume in predicting this and Bishop score was not shown to be a significant predictor.

Transvaginal cervical length measurement has been reported by several authors to provide a useful prediction of the likelihood of vaginal delivery within 24 h of induction of labour [4, 15–18]. According to the present study, a cervical length of less than 3.71 cm and cervical volume of less than 28.5cm3 were associated with a better chance of delivery within 24 h following labour induction. This value for the cervical length is different from the previously reported values; Pandis et al.,—28 mm, Gabriel et al.,- 26 mm and Tan et al., -20 mm [4, 16, 19].

The only available study that was done by Dilek et al. assessing cervical volume using two-dimensional (2D) ultrasonography at 22 weeks of gestation was to compare cervical volume and cervical length to predict preterm delivery in low-risk pregnancies [20]. They have concluded that cervical volume measurement by 2D ultrasound did not add any benefit compared with the cervical length measurement for the prediction of preterm birth [20]. Korean study done in 2012 assessed the place of cervical length and cervical volume to predict the onset of labour in women awaiting vaginal birth after caesarean [21]. It had been concluded that cervical volume was helpful in predicting the labour onset and cervical volume of fewer than 29.2 cm^3^ were more likely to experience a spontaneous onset of labour within the next 7 days [21]. A few studies have been published on the cervical volume using three-dimensional (3D) imaging [22–24]. A 3D imaging study done by Rovas et al. reported the value of cervical volume for predicting the onset of spontaneous labor at term [25]. In this study, it has been summarised that women with a higher vascularization index (VI) on 3D ultrasound had a higher chance of spontaneous onset of labour [25]. A study by Park et al. has concluded that combined screening using a short cervical length (≤ 28 mm) or a small cervical volume (≤ 20 cm^3^) may provide a better predictor for preterm birth [23]. Another study has shown that cervical length measurement was superior to cervical volume measurement assessed by 3D ultrasound for identifying women with increased risk of spontaneous preterm birth [24]. Therefore, there is an obvious paucity of studies assessing cervical volume measurement to predict the outcome of induction of labour at term and the present study has addressed this gap.

## Conclusion

Transvaginal sonographic measurement of cervical volume is significant in predicting the likelihood of vaginal delivery within 24 h of induction of labour. This has not reported previously. The commonly used sonographically measured cervical length is still more superior to the cervical volume in assessing this. Bishop score was not a significant predictor. The cervical volume of fewer than 27.6 cm^3^ may have a potential chance of delivery following induction of labour at term.

### Strengths and limitations

This study carries the initial work on the application of 2D ultrasound measurement of cervical volume to predict the outcome of induction of labour at term. The results may not be generalizable since the study was carried out in a single center and a small sample size needs to be acknowledged.

## Data Availability

Datasets generated from this study will be available from the corresponding author (MP) upon a reasonable request.

## Abbreviations

IOL: Induction of labour
NCTH: North Colombo Teaching Hospital
2D: Two-dimensional
ISUOG: International Society of Ultrasound in Obstetrics and Gynecology
TVS: Transvaginal ultrasound
NICE: National Institute of Clinical Excellence
ROC: Receiver Operating Characteristics
AUC: Area under the curve
SD: Standard deviation
IQR: Interquartile range
3D: Three-dimensional

## References

[1] Tsakiridis I, Mamopoulos A, Athanasiadis A, Dagklis T. Induction of Labor: an Overview of Guidelines. Obstet Gynecol Surv. 2020;75(1):61–72. 10.1097/OGX.0000000000000752 31999354

[2] Dhall K, Mittal SC, Kumar A (1987). Evaluation of preinduction scoring systems. Aust New Zeal J Obstet Gynaecol.

[3] Hughey MJ, McElin TWBC (1976). An evaluation of preinduction scoring systems. Obstet Gynecol.

[4] Pandis GK, Papageorghiou AT, Ramanathan VG, Thompson MO, Nicolaides KH (2001). Preinduction sonographic measurement of cervical length in the prediction of successful induction of labor. Ultrasound Obstet Gynecol.

[5] Sonek J, Shellhaas C (1998). Cervical sonography: a review. Ultrasound Obstet Gynecol.

[6] Boozarjomehri F, Timor-Tritsch I, Chao CR, Fox HE (1994). Transvaginal ultrasonographic evaluation of the cervix before labor: presence of cervical wedging is associated with shorter duration of induced labor. Am J Obstet Gynecol.

[7] Watson WJ, Stevens D, Welter S, Day D (1996). Factors predicting successful labor induction. Obstet Gynecol.

[8] Park KH, Kim SN, Lee SY, Jeong EH, Jung HJ, Oh KJ (2011). Comparison between sonographic cervical length and Bishop score in preinduction cervical assessment: a randomized trial. Ultrasound Obstet Gynecol.

[9] Kyo HP (2007). Transvaginal ultrasonographic cervical measurement in predicting failed labor induction and cesarean delivery for failure to progress in nulliparous women. J Korean Med Sci.

[10] Paterson-Brown S, Fisk NM, Edmonds DK, Rodeck CH (1991). Preinduction cervical assessment by Bishop’s score and transvaginal ultrasound. Eur J Obstet Gynecol Reprod Biol.

[11] Ezebialu IU, Eke AC, Eleje GU, Nwachukwu CE (2013). Methods for assessing pre-induction cervical ripening. Cochrane Database Syst Rev.

[12] Kagan KO, Sonek J (2015). How to measure cervical length. Ultrasound Obstet Gynecol.

[13] National Institute for Health and Clinical Excellence (2008). Induction of labour NICE clinical guideline 70.

[14] Editor (2014). Guideline on induction of labour 1. Sri Lanka J Obstet Gynaecol.

[15] Laencina AMG, Sánchez FG, Gimenez JH, Martínez MS, Martínez JAV, Vizcaíno VM (2007). Comparison of ultrasonographic cervical length and the Bishop score in predicting successful labor induction. Acta Obstet Gynecol Scand.

[16] Gabriel R, Darnaud T, Chalot F, Gonzalez N, Leymarie F, Quereux C (2002). Transvaginal sonography of the uterine cervix prior to labor induction. Ultrasound Obstet Gynecol.

[17] Saccone G, Simonetti B, Berghella V (2016). Transvaginal ultrasound cervical length for prediction of spontaneous labour at term: a systematic review and meta-analysis. BJOG.

[18] Verhoeven CJM, Opmeer BC, Oei SG, Latour V, Van Der Post JAM, Mol BWJ (2013). Transvaginal sonographic assessment of cervical length and wedging for predicting outcome of labor induction at term: a systematic review and meta-analysis. Ultrasound Obstet Gynecol.

[19] Tan PC, Vallikkannu N, Suguna S, Quek KF, Hassan J (2007). Transvaginal sonographic measurement of cervical length vs. Bishop score in labor induction at term: tolerability and prediction of cesarean delivery. Ultrasound Obstet Gynecol.

[20] Dilek TUK, Gurbuz A, Yazici G, Arslan M, Gulhan S, Pata O (2006). Comparison of cervical volume and cervical length to predict preterm delivery by transvaginal ultrasound. Am J Perinatol.

[21] Jo YS, Lee GSR, Kim N, Jang DG, Kim SJ, Lee Y (2012). Clinical efficacy of cervical length and volume for prediction of labor onset in VBAC candidates. Int J Med Sci.

[22] Jo YS, Jang DG, Kim N, Kim SJ, Lee G (2011). Comparison of cervical parameters by three-dimensional ultrasound according to parity and previous delivery mode. Int J Med Sci.

[23] Park IY, Kwon JY, Kwon JY, Hong SC, Choi HM, Kwon HS (2011). Usefulness of cervical volume by three-dimensional ultrasound in identifying the risk for preterm birth. Ultrasound Med Biol.

[24] Hoesli IM, Surbek DV, Tercanli S, Holzgreve W (1999). Three dimensional volume measurement of the cervix during pregnancy compared to conventional 2D-sonography. Int J Gynecol Obstet.

[25] Rovas L, Sladkevicius P, Strobel E, De Smet F, De Moor B, Valentin L (2006). Three-dimensional ultrasound assessment of the cervix for predicting time to spontaneous onset of labor and time to delivery in prolonged pregnancy. Ultrasound Obstet Gynecol.

